# Supplementation of Male Pheromone on Rock Substrates Attracts Female Rock Lizards to the Territories of Males: A Field Experiment

**DOI:** 10.1371/journal.pone.0030108

**Published:** 2012-01-13

**Authors:** José Martín, Pilar López

**Affiliations:** Departamento de Ecología Evolutiva, Museo Nacional de Ciencias Naturales, CSIC, Madrid, Spain; University of Wales Swansea, United Kingdom

## Abstract

**Background:**

Many animals produce elaborated sexual signals to attract mates, among them are common chemical sexual signals (pheromones) with an attracting function. Lizards produce chemical secretions for scent marking that may have a role in sexual selection. In the laboratory, female rock lizards (*Iberolacerta cyreni*) prefer the scent of males with more ergosterol in their femoral secretions. However, it is not known whether the scent-marks of male rock lizards may actually attract females to male territories in the field.

**Methodology/Principal Findings:**

In the field, we added ergosterol to rocks inside the territories of male lizards, and found that this manipulation resulted in increased relative densities of females in these territories. Furthermore, a higher number of females were observed associated to males in manipulated plots, which probably increased mating opportunities for males in these areas.

**Conclusions/Significance:**

These and previous laboratory results suggest that female rock lizards may select to settle in home ranges based on the characteristics of scent-marks from conspecific males. Therefore, male rock lizards might attract more females and obtain more matings by increasing the proportion of ergosterol when scent-marking their territories. However, previous studies suggest that the allocation of ergosterol to secretions may be costly and only high quality males could afford it, thus, allowing the evolution of scent-marks as an honest sexual display.

## Introduction

Many animals produce elaborated sexual signals, which in many cases are intended to attract potential mates [1]. Attraction of females to sexual signals of males may be mainly explained because these signals provide honest information about the characteristics of males [2], or because the signal exploits the sensory system of females that have a sensory bias for some traits [3], [4]. In any case, these male sexual signals can evolve by sexual selection to increase their attractiveness to females.

Research on sexual selection has often been biased towards studying animal signals that are visually conspicuous and attractive for humans too. Other sensory systems have received less attention [5], [6]. Chemoreception is, however, the main sensory system used by many animals, and chemical signals (pheromones) play an important role in the intraspecific communication and sexual selection of many types of animals, including vertebrates [5]–[7]. For example, in mammals, pheromones are frequently incorporated into feces, urine or other scent marks left on different substrates with the purpose of marking territorial boundaries or attracting mates [6], [8]–[10]. In addition, pheromones released in the water by some male fish [11], [12] and amphibians [13] may attract females and enhance male reproductive success.

Many lizards and snakes produce chemical secretions [14]–[17], which are often deposited in feces or substrate scent marks [17]–[20]. Behavioral tests have shown that chemicals in the scent or trailing marks of lizards and snakes may give information on sex, body size, age or familiarity recognition [14], [17], [21], or even provide more detailed information on morphological traits and health condition [22], [23]. This information seems important in intrasexual relationships between males [24]–[27] and in female mate choice [28]–[31]. Laboratory tests suggest that female lizards might use some chemicals found in the scent marks of males as honest signals to select areas scent marked, and, thereby, occupied by preferred potential mates [29], [30]. On the other hand, a pre-existing sensory bias for food chemicals might also explain the chemosensory preferences of female lizards for some chemicals in the scent of males [32].

Consequently, male lizards might use scent marks to attract females to their territories, thus, increasing the probabilities of mating with these females. But this attracting function of the scent marks of lizards remains largely untested. Furthermore, some field studies suggest that female lizards might choose to establish in an area based just on microhabitat or thermal characteristics, abundance of food or refuges, etc. [33]–[36]. Thus, females might base their space use on the quality of a territory rather than on sexual signals informing on the characteristics and quality of the male that defends that territory, as found in other animals [37]. Males would only defend these favorable territories from other males to increase their access to females [33], [34], [38]. Nevertheless, it is still possible that females might be attracted to a territory through being “lured” by male signals that resemble food [4], [32] or by male signals that may be used as “public information” to assess the quality of a territory [39], [40].

The Carpetan rock lizard, *Iberolacerta cyreni* (formerly *Lacerta monticola cyreni*), is a small diurnal lacertid lizard found in rocky mountain habitats of the center of the Iberian Peninsula [41]. This is a polygynandrous species, where older (larger) males defend territories that partly overlap with those of other males [42], and where few males obtain most of the successful matings, siring the offspring of several females [43]. Males scent mark substrates with secretions from the femoral glands, which contain proteins and lipids such as fatty acids and steroids [22], [44]. In the laboratory, female *I. cyreni* discriminate and show strong chemosensory responses to ergosterol, or to the scents of males that allocate a higher proportion of this steroid to femoral secretions [29]–[30], [45]. These males are those of presumably high quality (i.e., those more symmetric and with a higher immune response), which suggests that females may use this compound to choose potential mates of high quality [29].

More interestingly, in a laboratory terrarium, female rock lizards prefer to use areas that were experimentally manipulated to increase the proportion of ergosterol in the scent marks of males [29]. This result suggests that chemosensory preferences of females for male signals affect their space use, and, more importantly, that these changes may increase the opportunities of a female for mating with the male that has scent marked a selected particular area. However, most of these studies were made under laboratory conditions where it is difficult to evaluate the actual importance of observed lizard behaviors for mating success in the field. Nevertheless, paternity data from a field study suggest that females move around and select to mate with a few specific males [43].

In this paper, we designed a field experiment to simulate the presence of scent marks of male rock lizards (*I. cyreni*) of presumably high quality. We experimentally added ergosterol to rocks inside home ranges occupied by male lizards, and examined the effects of this manipulation on the observed density of females in that area. We predicted that if these “pheromone-enhanced” scent marks signaled the presence of males of higher quality to females [29], or if females had a sensory bias for this chemical [32], the experimental areas where we increased ergosterol on rocks should be more attractive for females. Therefore, this manipulation should result in an increase in the density of females occupying these areas, and in a subsequent increase of mating opportunities for males living in these areas.

## Materials and Methods

### Study site and experimental plots

We conducted the field study during May–June 2009 at “Alto del Telégrafo” (Guadarrama Mountains, Madrid Prov., central Spain) at an elevation of 1,900 m. Granite rock boulders and screes interspersed with shrubs (*Cytisus oromediterraneus* and *Juniperus communis*) predominated at the study site, together with meadows of *Festuca* and other grasses [46], [47]. In this area, lizards are active from late April to early October, mating in May–June and producing a single clutch in July [43].

We performed the field experiment on a large mountain slope oriented to the south where *I. cyreni* lizards were abundant, and where the habitat and microclimate were homogeneous. In this zone, we selected 12 rectangular areas (15×6 m each) that were separated by at least 25 m. Inside each area, we used color flags to mark two plots (2×2 m each), the centers of which were separated by 7 m. Each plot was selected to include a high cover of large rocks and some bushes, like the microhabitats selected by lizards [46], [47]. We performed the experimental supplementations within these plots. One of the plots within each area was randomly assigned to the experimental treatment and the other was assigned to the control treatment. These areas were not switched between days (i.e., the same plots designated as experimental in the first day were used as experimental during all observations). We preferred this approach instead of randomizing to allow that lizards could move through both areas and finally select to settle in some areas, thus, allowing a cumulative effect through time. We considered that it was very unlikely that lizards would switch frequently between territories once they had been established, which would confound the results if we changed the location of the treatments every day.

We recorded microhabitat structure to ensure that experimental and control plots were homogeneous. We noted the presence and types of vegetation and different substrates at different heights on four 1 m transects [46], [47]. Results of General Linear Models (GLMs) showed that there were no significant differences for any habitat variable (dependent variables) between the control and experimental plots (paired within each area as a repeated measures factor) (0.14<*F*_1,11_<0.71, 0.22<*P*<0.70 for all variables).

### Manipulation of scent marks

We made the experimental supplementation during four separate days (27th May, 2nd June, 3rd June and 11th June) with sun and temperature conditions that allowed lizards to be fully active. We initially intended to perform the experiment on alternate days, but bad weather conditions at the high mountain altitude limited the activity of lizards entirely or to a great amount, which meant we could only perform the experiment during these four days.

We prepared two liquid solutions (experimental and control) on the same days as the tests. For the experimental solution, we filled clean dark glass bottles with dichloromethane (DCM) and dissolved ergosterol in it (authentic standard; both compounds were GC grade, from Sigma-Aldrich Chemicals) in a proportion of 25 g ergosterol/1 L of DCM. In a previous study, female rock lizards showed high chemosensory responses (i.e., high tongue-flick rates) to cotton swabs impregnated with this concentration of ergosterol [29]. These responses were only slightly higher than average responses to natural femoral secretions of males [29]. The control solution was DCM alone treated similarly and kept in similar bottles. The DCM alone elicits very low chemosensory responses in female rock lizards [29]. Then, we mixed the solutions with a vortex, and kept all the bottles in a portable refrigerator to transfer them to the field.

In the early morning (from 06.30 h to 07.30 h, GMT), before lizards were active, we used different painting brushes to impregnate rocks with the experimental solution or the control one within the designated plots. We used 100 cL of the appropriate solution for each plot to impregnate some areas of all large and medium sized exposed rocks present within each 2×2 plot. We intended to simulate the scent marks of males, by using a small brush to impregnate small selected rectangular areas (about 15×5 cm), haphazardly distributed along the rocks, but we specially impregnated locations on the tops of high rocks or close to refuges (rock crevices). We selected these areas because lizards are known to deposit fecal pellets and scent marks in these particular locations [18], [48], and this is where chemosensory exploration of substrates by tongue-flicking seems to be more intense (pers. observ.). Immediately after setting out the experimental solution, the DCM readily evaporated leaving the ergosterol deposited on the rocks. The control solution evaporated entirely without leaving any solid residue.

### Estimation of lizard densities

To estimate the density of lizards we made seven counts of lizards observed in the control and experimental plots every day, and calculated average numbers of lizards observed in each plot per census in each day. We made five estimates of lizard densities in five different days: the first estimate was made three days before starting the experiment (to obtain initial densities), with a subsequent estimate being made on each of the four days immediately after we manipulated rocks. Although this method only provided estimations of relative densities, we preferred this non-invasive method to a mark-recapture study, which may have affected the normal behavior of lizards. Because we studied immediate responses (space use) of lizards to the manipulation in a given day, the effort needed to capture lizards during a day would not have allowed us to observe their normal space use behavior in response to the chemical manipulation in that day. Also, because the mating season of these lizards is very limited in time (only a few days immediately after emerging from hibernation), we could not simultaneously perform a mark-recapture study with the observations of natural responses of lizards to the scent manipulation.

During this study, lizards emerged from night refuges around 07.30 h (GMT), when environmental temperatures were appropriate, and lizards started basking on rocks to achieve optimal body temperatures before moving around for foraging or looking for mates. So we started census of lizards at 08.00 h. We made seven censuses every day, one every 30 min, until the hot midday temperatures started to lower the activity of lizards. In each census, one experimenter quietly approached one plot and stopped at a distance of about 5 m. From several points located at this distance surrounding the plot and observing with binoculars, we were able to achieve a total vision of all points in the plots where lizards could be active. Our censuses did not disturb the behavior of lizards, which did not show alarm or escape responses to our presence and continued with their normal activities during the observations. We recorded the numbers of lizards observed active within the experimental and control plots and in a 1 m area surrounding each plot (a total area of 4×4 m per plot). Based on the size and coloration of lizards, we classified individuals as adult males (with dorsal green coloration and large heads), adult females (with dorsal brown coloration and small heads), and non-reproductive subadults (brown coloration and clearly smaller in body size; i.e., SVL<60 mm) [41], [42]. When several individuals were observed in the same census, the simultaneous observation, or the differential characteristics, of different individuals easily allowed the number of different individuals to be easily estimated in one census.

Because lizards moved frequently through their home ranges, we considered that lizards could be aware of the manipulations of the two plots in each area, and that observations of lizards close to the control or experimental plots (within the 4×4 m area surrounding the centre of each plot) could reflect a preference of lizards for using microhabitats within or close to a particular plot. In comparison with natural home ranges of this lizard species [42], [43], the surveyed area (4×4 m) could be similar in size to the natural core area of a lizard's home range (i.e., the most exclusive and used locations based on a density function of sightings, which is around 4 m^2^ for females and 18 m^2^ for males), while the total size of a natural home range (i.e., averages between 75–150 m^2^ for males and 28–50 m^2^ for females) would allow a lizard to explore both plots in an area. To standardize search effort in all observations, we spent 5 min watching each pair of plots before moving to another pair of plots.

For each plot, we first calculated the daily average number of lizards observed per census (adult males or females or subadults) from the seven censuses made in each day. We used GLMs to examine variations in the daily average number of lizards observed in a census per plot (square root transformed because this was a count variable) between treatments (control vs. experimental plots paired within each area) and days (one initial day before starting the experiments, and the four days of the manipulation), both as repeated measures factors. We included the interaction in the model to test whether differences between the control and experimental plots varied between days. Post hoc pairwise comparisons were based on Tukey's honestly significant difference (HSD) tests [49].

### Association of females with males

To assess the effect of the manipulation on potential mating opportunities for males, during the censuses we noted the number of females that were observed associated in close proximity (less than 50 cm) to a male [50]. In this and other lizard species, a male close to a female could obtain a copulation with that female because even if the female would not accept it, the male might try to obtain a forced mating. However, under the mate attraction hypothesis, because we manipulated the characteristics of the territories, but not the characteristics of the males found there, we did not expect that females accepted matings from any male found in the plot, but only if the male was “congruent” with that expected by females based on their sexual chemical signals in scent marks (i.e., visual signals would confirm the honesty of the chemical signal). Thus, we did not record the number of actual matings observed, as these were not considered representative of the normal behavior of lizards outside of the experimental conditions.

We used a two-tailed binomial test to compare the number of females found associated to a male in the control and experimental plots, and used a chi-square test to compare the proportions of males observed alone or close to females in the control and experimental plots. Although observations of the same individual lizards could occur in different days, this would not affect the results, because multiple matings with the same or different females will increase the mating success of a male, given that multiple paternity occurs in this species [43].

## Results

### Effects of scent manipulation on lizard density

All plots were occupied by some lizards in at least some of the census. The total number of lizards observed in one plot in one single census ranged between 0 and 10 individuals (mean±SE = 0.42±0.03 lizards; males: 0.19±0.02; females: 0.16±0.02; subadults: 0.08±0.01).

**Figure 1:**
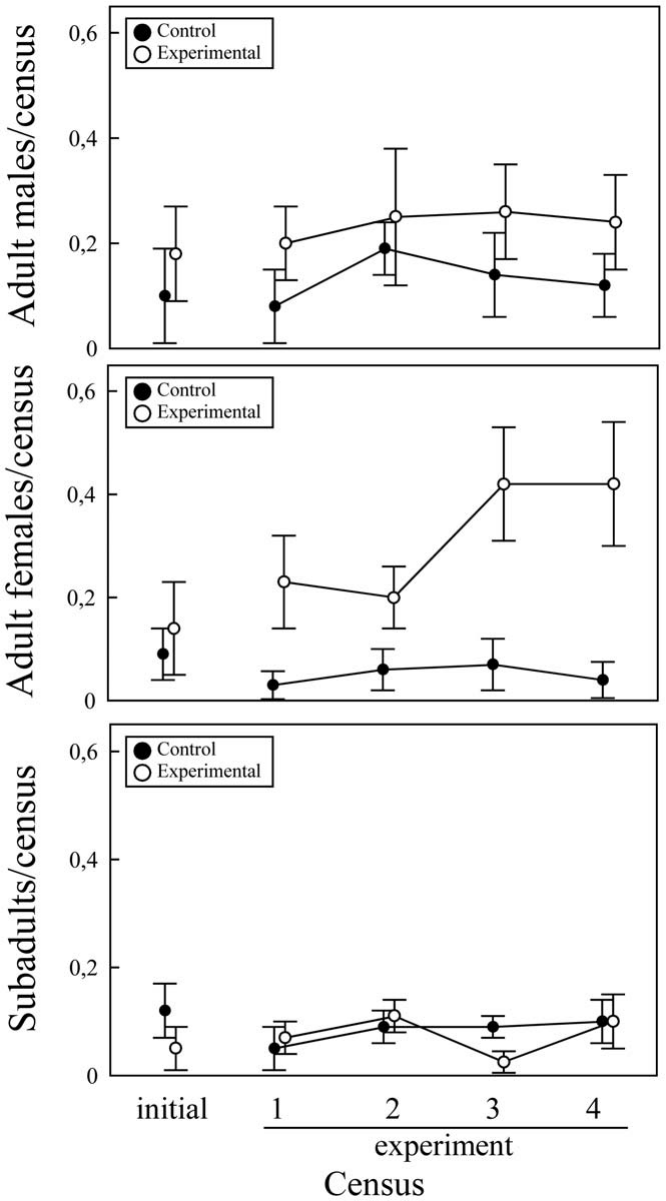
Effects of scent manipulation on lizard density. Mean±SE number of (a) adult males, (b) adult females or (c) subadult lizards observed in each census of the control (black circles) and experimental (open circles) plots before the experiment (initial) and during the four days after rocks were supplemented with ergosterol (experimental) or a control solution.

The average number of adult males per census did not significantly differ between sampling days (GLM, day: *F*_4,44_ = 0.62, *P* = 0.65) nor between the control and experimental plots (treatment: *F*_1,11_ = 3.12, *P* = 0.10) and the interaction was not significant (*F*_4,44_ = 0.35, *P* = 0.84) (Fig. 1a). Therefore, the experimental manipulation did not affect the observed densities of males.

In contrast, the effect of our treatments on the average number of females observed in the different plots depended on sampling day (GLM, day: *F*_4,44_ = 1.28, *P* = 0.29; treatment: *F*_1,11_ = 3.36, *P* = 0.09; interaction: *F*_4,44_ = 2.83, *P* = 0.037) (Fig. 1b). Thus, although the control and experimental plots did not significantly differ in the number of females observed in the initial census previous to the experiment (Tukey's tests: *P* = 0.96), or in the first (*P* = 0.43) and second day of the experiment (*P* = 0.99), there was a significantly higher number of females in the experimental plots in the third (*P* = 0.007) and fourth day (*P* = 0.017).

With respect to subadult lizards, the average number observed per census did not significantly differ between sampling days (GLM, day: *F*_4,44_ = 1.03, *P* = 0.40) nor between the control and experimental plots (treatment: *F*_1,11_ = 0.39, *P* = 0.54) and the interaction was not significant (*F*_4,44_ = 1.06, *P* = 0.39) (Fig. 1c).

### Association of females with males

**Figure 2:**
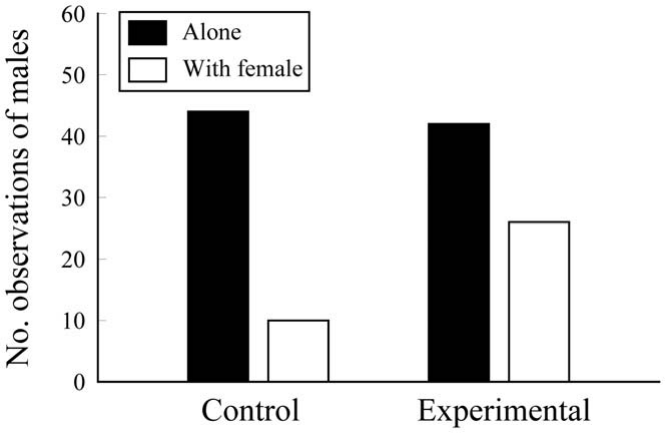
Association of females with males. Observations of adult male lizards that were alone (black bars) or close (less than 50 cm) to one or several adult females (open bars) in the control and experimental plots.

During the censuses made after the experimental manipulations, we observed 46 females associated in proximity to a male in the experimental plots and only 10 females close to a male in the control plots (two-tailed binomial test, *P*<0.0001). Thus, males were found associated to one or more females in 38.2% of observations of males in experimental plots and only in 18.5% of observations in control plots (χ^2^ = 5.62, *P = *0.018, d.f. = 1) (Fig. 2). These results suggested that potential mating opportunities for males were greater in experimental plots.

## Discussion

Our experimental field study showed that increasing ergosterol on rock substrates used by male *I. cyreni* lizards in their home range areas resulted in increased relative densities of females in those areas. This result confirms the observations from previous laboratory experiments indicating that female *I. cyreni* may modify their space use to increase the use of areas where substrate scent-marks have more ergosterol [29]. Moreover, the current study showed that increased densities of females in experimental plots might effectively result in an increase of mating opportunities for males that inhabited in these territories because we observed more females close to males in experimental plots. Therefore, male rock lizards might potentially attract more females and obtain more matings by increasing the proportion of ergosterol when scent-marking their territories.

When selecting where to establish a home range, an individual must consider several factors such as physical ones (e.g., temperature, humidity) or the availability of biotic resources (e.g., food, potential mates, absence of predators) [51], [52]. In many animals, conspecific cues, very often chemical cues, are used as signals of habitat quality, indicating the presence of food, good environmental conditions, or low predation risk [53]–[57]. Hence, individuals may settle in an area because they are attracted to conspecific cues, rather than to habitat features. For example, male rock lizards select refuges based on the chemical cues of conspecifics, possibly because this is a cue of a refuge free of snake predators [56].

Moreover, the public information theory proposes that not only the presence but also the performance of individuals might serve as a cue for habitat assessment [39], [40]. For example, before natal dispersal, juvenile common lizards, *Lacerta vivipara*, use social information, through conspecific chemical cues, to decide settling in a home range [57]. Similarly, male rock lizards use chemicals in feces and scent marks of conspecifics to decide whether to enter a home range used by other males by evaluating their relative competitive ability (e.g., body size differences) [18], [48], [58]. Our current study and previous laboratory results suggest that female rock lizards may also choose to settle in a home range based on characteristics of chemical cues in scent marks from males. In our field experiment, both the microhabitat characteristics and the initial densities of male and female lizards in each pair of plots were similar, and males did not seem to modify their space use during the experiment. Thus, we could discard that females selected experimental plots by specific characteristics of the habitat or by the actual presence or distribution of a higher number of potential mates. Therefore, only the higher proportion of ergosterol in scent marks should explain why the density of females increased in experimental plots.

There are several alternative explanations about why female rock lizards are attracted to areas with more ergosterol on the substrate. Females might directly use this male trait (i.e., higher levels of ergosterol in scent marks) as a reliable advertisement of the quality of a male, and use this signal to increase the possibility of mating with the male that has scent marking that area. In fact, there is a relationship between “quality” of a male rock lizard and the proportion of ergosterol in their femoral secretions [22], [29]. Also, in other animals, the characteristics of pheromones seem to be affected by the ‘quality’ and health state of the male [7]. Theoretical models of mate choice without direct benefits predict that sexual signals can only be evolutionarily stable if they are honest and condition dependent or costly to the signaler and if the cost is correlated with the signaler's quality [2], [59], [60]. Chemical signals of lizards may be honest because the allocation of ergosterol, or other compounds, to secretions is costly and dependent on the ability of a male to obtain a good quality diet [30], [61]. Also, there may be a trade-off between the physiological regulation of the immune system and the allocation of essential nutrients (e.g., vitamins or other ‘costly’ lipids) to sexual chemical ornaments [62]. Therefore, only males in good condition could mount a strong immune defense and produce an extravagant sexual ornament [63]–[65].

Alternatively, females might be attracted to ergosterol per se because this could be a food stimulus indicating the presence of food, and females might have a sensory bias for this chemical food stimulus independen of the male signal, as has been suggested in a previous experiment with this lizard species [32]. Similarly, insectivorous *Liolaemus lemniscatus* lizards stay for longer and do more chemical exploration in areas where chemical cues from mealworms are present [66]. Females of other animals, such as some moths or crickets, may also be attracted to food chemicals provided by males of their own species in their pheromones or nuptial food gifts [67], [68]. Nevertheless, if the allocation of this chemical to femoral secretions was costly and only high quality males could afford it, a pre-existing sensory bias for essential nutrients might further allow the evolution of an honest sexual display [4], [32], [69].

In addition, it might be possible that females used the “quality” of the scent marks of males to estimate the quality of a territory per se, and not the characteristics of the male that has scent marked it. Males of higher quality are predictably those that may control the highest quality territories with respect to the availability of food, refuges or thermal resources [70]. It was suggested that female lizards *Uta stansburiana* might, for example, assess male body size as an indicator of the thermal quality of territories [35]. Female lizards might use not only the presence of these high quality males, but also the information provided by their chemical cues in scent marks, to choose territories of high quality. This strategy might be favored by female rock lizards because males emerge from hibernation earlier than females, and fight for territories with other males [42], [43]. Thus, when females start to look for suitable home ranges, the chemical signals of males could be a good/quick cue of territory quality with respect to resources needed by females other than the availability of potential mates. Nevertheless, females might also benefit by increasing the probability of mating with the high quality males that should “normally” defend these high quality territories.

In other lizard species, observations and experimental manipulations of the quality of male territories have suggested that females select the quality of the territory (e.g., electing better food or thermoregulatory opportunities) rather than the quality of the male that defends this territory [33], [35], [36]. However, some of these studies also show strong female preferences for some males of higher quality (e.g., for large males) by investing more in current reproduction and individual progeny when mating with these males [35]. In nature, females that selected high quality territories may expect to find high quality males as well. However, some of the characteristics that allow males to defend a territory from other males may not be attractive for females [71]. Thus, a female should also exert some mate choice criteria. This would explain why female rock lizards do not always prefer the scent marks of dominant males, but select other characteristics of males [72]. In addition, a field study showed that often the males that father the offspring of females are not always those that live close to a female's home range, but only a few specific males that obtained most of the matings [43].

The responses of female rock lizards to the experimental manipulation were not observed until the third day after the beginning of the supplementation of ergosterol. This could be explained simply because females that lived in the vicinity of the plots needed some time before they explored and were aware of the “potential high quality” of the experimental plots, and decided to move to these areas. But it also might indicate that females needed a reinforcement of the signal during repeated days to ensure that the signal was reliable. This is because any male might potentially invest in producing small amounts of femoral secretions with a higher proportion of ergosterol attractive for females. However, this strategy would be only useful for scent marking during a single day because the environmental conditions may quickly degrade scent marks [73]. Only high quality males may produce larger quantities of secretions with a higher proportion of ergosterol being required to scent mark and remark their territories over several consecutive days. The amount of deposition may convey information about the physiological condition that supplements, and confers reliability to, the information conveyed by the chemical structure of the chemical signal [20]. Finally, under the public information hypotheses [39], [40], it is possible that females also use the presence of other females in a territory, or their chemical cues, as an additional index of quality of that territory, resulting in accumulative numbers of females in favorable plots through successive days.

Although our experimental manipulation affected the densities of available females, it did not seem to affect space use of males. This result suggests that the information provided by ergosterol in scent marks might not be important for males when deciding their space use. In fact, previous studies showed that male rock lizards have low chemosensory responses to this chemical, which, in contrast, elicited higher responses in females. However, the converse occurs for other chemicals (i.e., those related to male body size) that may affect intrasexual relationships and agonistics contests between males [26], [45]. If the density of females increased in experimental plots, we might expect that males also moved to experimental plots looking for females. Something similar occurred when female densities were experimentally manipulated in some lizard species [74] but not in others [33], [75]. These interspecific differences were explained because the males of long lived species, such as rock lizards, may maintain territories to maximize long-term reproductive success [74]. It is likely that it was too costly for males to leave an already owned territory and look for potential females in the territories of other males. Nevertheless, although highly unlikely, we cannot exclude the possibility that our treatment affected male quality (i.e., the better males went to the experimentally treated plots and replaced others), which might also increased the attraction of females to these high quality males and not because of the experimental manipulation of these territories.

Densities of subadult, non-reproductive lizards did not change after the experimental manipulation. This may suggest that these young lizards did not use scent marks of males to select home ranges (even if these scent marks might inform of the quality of a territory). Alternatively, these younger lizards may be unaware of the manipulation because they do not wander widely across home ranges [42], and there may also be costs of moving to unknown areas, such as receiving more aggression from non familiar territorial adult males with which social relationships have not been previously established [58], [76], [77], or incurring high predation risk during dispersal through unknown areas.

We conclude that, as in many other animals [5], [10]–[12], some chemicals in the scent marks of male lizards may function as pheromones that attract females and enhance the reproductive success of males. Male rock lizards may attract more females and obtain more matings by increasing the proportion of ergosterol when scent-marking their territories. However, the allocation of ergosterol, or other chemicals with pheromonal activity, to secretions may be costly and only high quality males could afford it, thus, allowing the evolution of pheromones and scent marks as an honest sexual display.
